# Predation scars provide a new method to distinguish native and invasive crab predation on mollusc prey

**DOI:** 10.1002/ece3.70338

**Published:** 2024-09-23

**Authors:** Kristina M. Barclay, Paige Amos, Lindsey R. Leighton, Chris L. Schneider, Julia K. Baum

**Affiliations:** ^1^ Department of Biology University of Victoria Victoria British Columbia Canada; ^2^ Department of Anthropology University of Victoria Victoria British Columbia Canada; ^3^ Department of Earth and Atmospheric Sciences University of Alberta Edmonton Alberta Canada

**Keywords:** alternative data, data‐poor fisheries, discriminant function analysis, invasive species, relative warp analysis, repair scar

## Abstract

Crab species are increasingly important socioeconomic resources that are threatened by human exploitation, climate change, and invasive species, such as European green crabs (*Carcinus maenas*). However, the continued health of their populations is often uncertain given the limited long‐term population data, necessitating alternate approaches to ensure their continued viability. Furthermore, *C. maenas* are one of the most highly invasive and destructive marine species globally, posing a threat to local ecosystems and species, including socioeconomically important crabs and their mollusc prey. Improved understanding of *C. maenas* invasions and their impacts on local crab and mollusc resources is therefore vitally important. Here, we present a new method for identifying species‐level presence and relative abundances of important crab species, including invasive *C. maenas*, from the scars they leave on their prey. We conducted controlled manipulative feeding experiments in which individuals of Dungeness crabs (*Metacarcinus magister*), red rock crabs (*Cancer productus*), and *C. maenas*, were allowed to attack snails (*Tegula funebralis*) and produce sublethal shell damage. Resulting shell damage was photographed and landmarked for geometric morphometric analyses to determine any differences in the shape of shell damage between crab species. There were statistically significant differences between the shape of shell damage created by all three crab species (*p* < .0001). Shell damage formed a gradient from narrow/deep (*C. productus*) to shallow/wide (*C. maenas*) with *M. magister* as an intermediate form. Our method provides a novel, cost‐effective tool for long‐term species‐specific reconstructions of crab populations and assessing the broader ecological impacts of *C. maenas* invasions that can inform management and mitigation for these three important crab species.

## Graphical abstract

We present a new cost‐effective method for identifying species‐specific crab predation on mollusc prey. By applying geometric morphometrics to analyse the shape of the damage they leave on their shelled prey, we quantitatively demonstrate that each species of crab, including native Dungeness and red rock crabs, as well as invasive European green crabs, produces uniquely shaped damage. Our method can be used to reconstruct species‐specific crab abundances and help understand the ecological implications of European green crab invasions on native crabs, mollusc prey, and their broader ecosystems.

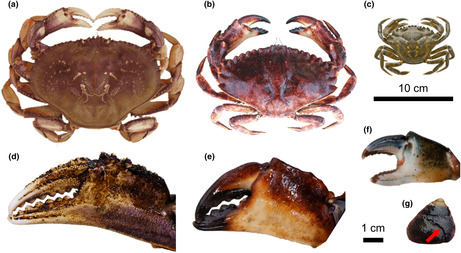

## 1 INTRODUCTION

European green crabs (*Carcinus maenas*) are voracious shell‐crushing predators and a highly successful invasive species that threaten many coastal species and ecosystems (Ens et al., 2022; Young & Elliott, 2020), including native crab species (Behrens Yamada et al., 2010; Colautti et al., 2006; Ens et al., 2022; Hunt & Behrens Yamada, 2003; McDonald et al., 2001), socioeconomically important molluscs (Colautti et al., 2006; Ens et al., 2022; Grosholz et al., 2011), and critical eelgrass habitats (Ens et al., 2022; Garbary et al., 2014; Howard et al., 2019; Malyshev & Quijón, 2011; Neckles, 2015). On the west coast of North America, *C. maenas* were first reported from San Francisco Bay in 1989 and have now expanded as far north as Haida Gwaii, British Columbia as of 2020 (Council of the Haida Nation, 2020; Ens et al., 2022) and Annette Island, Alaska as of 2022 (NOAA Fisheries, 2022). Despite costly ongoing mitigation efforts, *C. maenas* have proven incredibly difficult to eradicate (Ens et al., 2022; Green & Grosholz, 2021; Tummon Flynn et al., 2024) and have well‐established populations from California to British Columbia (Ens et al., 2022; Young & Elliott, 2020). *C. maenas* also negatively impact native crab populations through competition, predation of juveniles (Behrens Yamada et al., 2010; Ens et al., 2022; Jamieson et al., 1998; McDonald et al., 2001), and the destruction of important nursery habitats, such as seagrass meadows (Howard et al., 2019; McMillan et al., 1995). The ecological consequences of these negative interactions, as well as the impacts of *C. maenas* predation on native species, including impacts on commercially important bivalves, are a major concern for coastal communities and resource managers (Ens et al., 2022; Grosholz et al., 2011; Young & Elliott, 2020). While native red rock crabs (*Cancer productus*) appear to exert some level of control over *C. maenas* via predation and competitive exclusion (Hunt & Behrens Yamada, 2003; Jensen et al., 2007), the widespread implications of these interactions are not fully understood. Therefore, any information that can improve understanding of the long‐term impacts of *C. maenas* invasions on ecosystems and socioeconomically important species, such as native crab species and their mollusc prey, or provide a means to aid in the costs or efficiency of *C. maenas* mitigation/management, is vital.

Like other species, crabs and their ecosystems face ongoing threats from climate change, including ocean acidification, warming, hypoxia, and related harmful algal blooms (Alin et al., 2023; Bednaršek et al., 2020; Berger et al., 2021; Fisher et al., 2021; Froehlich et al., 2017), as well as from overfishing (Ban et al., 2017; Fitzgerald et al., 2018, 2019; Frid et al., 2016), and competition from invasive *C. maenas* (Behrens Yamada et al., 2010; Colautti et al., 2006; Ens et al., 2022; Hunt & Behrens Yamada, 2003; McDonald et al., 2001). Despite these mounting pressures, commercial crab fishing along the west coast of North America has rapidly expanded in recent decades (Ban et al., 2017; Boenish et al., 2021; Fisheries and Oceans Canada, 2024; Fitzgerald et al., 2019; Frid et al., 2016) to the point where most legal‐sized males are caught each season (Froehlich et al., 2017). These expansions have led to growing concerns over the continued sustainability of their populations and management (Barclay & Leighton, 2022; Fitzgerald et al., 2019; Froehlich et al., 2017; Helliwell, 2009), as well as Indigenous communities' access to crabs along the west coast of Canada and the United States (Ban et al., 2017; Fisher, 2023; Frid et al., 2016). Current management strategies for crab fisheries include size limits and sex restrictions (male only), as well as a limited number of commercial crab vessel licences in several management zones in British Columbia (Fisheries and Oceans Canada, 2024). Crabs are also typically managed as multi‐species fisheries (Culver et al., 2010; Fisheries and Oceans Canada, 2024; Fitzgerald et al., 2018), yet *Metacarcinus magister* are the primary target of commercial crab fisheries. The effectiveness of current management strategies is poorly constrained given the very limited historical records of crab fisheries (Culver et al., 2010; Fitzgerald et al., 2018, 2019; Helliwell, 2009). Instead, alternative data should be more frequently incorporated into species management (McClenachan et al., 2012). For example, firsthand accounts from Indigenous community members have demonstrated that commercial crab fishing has had negative impacts on crab populations over the last several decades (Ban et al., 2017).

An alternative data source that could be used to extend long‐term records of crab populations as far back as the fossil record are the predation scars left on mollusc prey by crabs. Crabs create distinct wedge‐shaped damage to prey by breaking prey shell margins with their claws (Boulding, 1984; Stafford, Dietl, et al., 2015; Teck et al., 2023; Vermeij, 1982a, 1982b), providing a record of all sublethal predation attempts experienced over the course of a prey's lifetime. These scars, known as repair scars, are widely used by palaeontologists to study shell‐crushing predation through time (Alexander & Dietl, 2003; Dietl & Kosloski, 2013; Leighton, 2002; Mondal et al., 2014; Mondal & Harries, 2015; Pruden et al., 2018; Richards & Leighton, 2012; Schindel et al., 1982; Vermeij, 1982b, 1983; Vermeij et al., 1981). Repair scars have also been used to study shell‐crushing predation in modern systems (Cadée et al., 1997; Geller, 1983; Molinaro et al., 2014; Schindler et al., 1994; Stafford, Tyler, & Leighton, 2015; Teck et al., 2023; Tyler et al., 2019; Vermeij, 1982a; Whitenack & Herbert, 2015), where they have been shown to be a good proxy for assessing relative crab abundance (Molinaro et al., 2014; Stafford, Tyler, & Leighton, 2015). For example, the frequency of repair scars on prey is greater in wave‐sheltered sites compared to wave‐exposed sites (Molinaro et al., 2014), tracking surveys of crab abundance (Stafford, Tyler, & Leighton, 2015). Repair scars have also been applied to study crab abundances, with population declines reported for southern California compared to the Pleistocene (Barclay & Leighton, 2022). However, while one other study has observed differences in the shape of shell damage created between crab species in the Atlantic (Dietl et al., 2010), there have been no attempts to test whether scar shape can be used for species‐level identifications of crabs, particularly invasive *C. maenas*.

Geometric morphometrics is a common and useful method for quantitatively evaluating changes in shape between organisms or features (Webster & Sheets, 2010) but has not been applied to studies of repair scar shape between crab species. In landmark‐based geometric morphometrics, morphological features of interest are denoted by a series of analogous points (landmarks or semi‐landmarks), allowing for spatial comparison of features and/or overall shape change between specimens or groups (Webster & Sheets, 2010). As such, geometric morphometric analyses provide a concrete method for quantifying variation in shape between specimens or groups that could easily be applied to assess differences in the repair scars created by different crab species.

Our goal here was to determine if the shape of predatory crab claw marks left on their prey could be used as a method to identify and monitor the presence and abundances of individual crab species, including *C. maenas*, as well as the ecological impacts of their predation on shelled prey species. We present a novel and simple method that allows determination of species’ presence and relative abundance for three important northeastern Pacific crab species, *M. magister*, *C. productus*, and invasive *C. maenas*, based on the shape of the predation scars they leave on a common prey item. We use geometric morphometrics to assess the shape of shell damage created on a common prey item by these three crab species under experimental conditions. This cost‐effective, easily reproducible method has the potential to be used to detect possible *C. maenas* invasions, abundances, and impacts to prey species, and to reconstruct species‐specific crab population abundances through time to aid in their continued sustainability.

## 2 MATERIALS AND METHODS

### 2.1 Crabs

**FIGURE 1:**
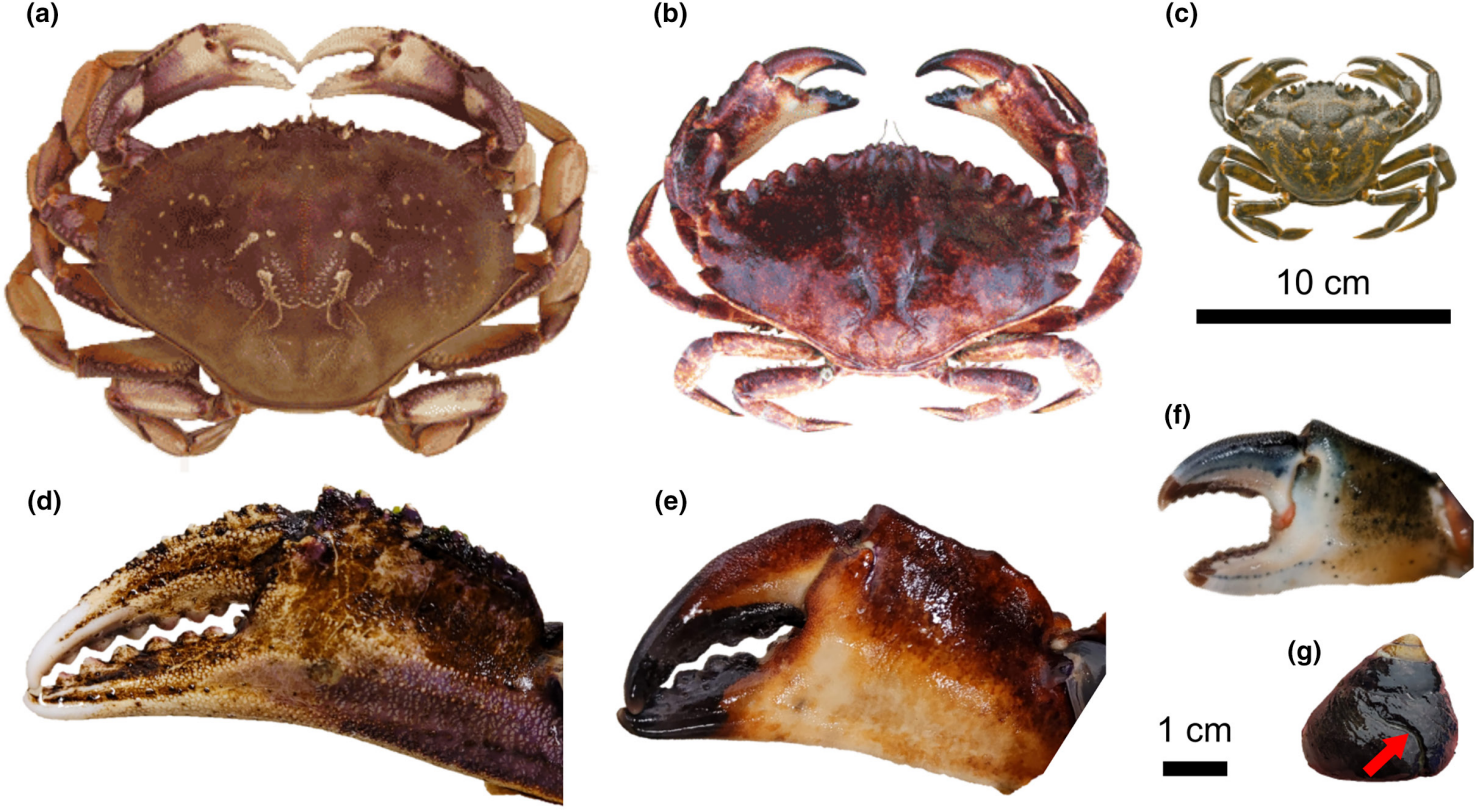
Images of study species used in experimental trails. Adult male crabs and claws of *Metacarcinus magister* (a, d), *Cancer productus* (b, e), and *Carcinus maenas* (c, f) and the prey species, *Tegula funebralis* with a red arrow denoting a repair scar from a healed crab attack (g). The 10 cm scale bar pertains to images of whole crabs (a–c), and the 1 cm scale bar pertains to images of crab claws and *T. funebralis* (d–g).

Individuals of three crab species (*M. magister*, *C. maenas*, *C. productus*; Figure 1) were wild caught around the southern end of Vancouver Island in 2022 and acclimated to laboratory conditions over at least 1 month prior to experiments. Prior to use in experiments, crabs were put on a reduced feeding schedule (once a week for *M. magister* and *C. productus*, three times a week for *C. maenas*) to increase hunger and interest in prey offered during the experiments. Crabs were uniquely labelled using numbered wire markers affixed to the right anterior side of the carapace. For each crab, maximum carapace width as well as left and right claw heights (height of the propodus directly behind the dactyl) were measured using a digital callipers (±0.01 mm; Table 1).

**TABLE 1:** Size of adult male crabs of *Metacarcinus magister*, *Carcinus maenus*, and *Cancer productus* used in feeding experiments.

Species	Crab ID	Carapace width (mm)	Left claw height (mm)	Right claw height (mm)	Snails attacked
*Metacarcinus magister*	D1	191.71	38.51	37.83	5, 3, 6, 4
*Metacarcinus magister*	D2	194.21	38.71	39.12	10, 11
*Metacarcinus magister*	D3	205.54	40.61	39.92	1, 7, 8, 9, 2
*Carcinus maenas*	EGC1	75.6	19.39	25.05	28, 29, 30, 31, 32
*Carcinus maenas*	EGC5	76.41	19.59	26.78	19, 20, 21
*Carcinus maenas*	EGC6	74.66	19.39	26.78	24, 25, 26, 27
*Carcinus maenas*	EGC10	80.2	27.84	20.55	17, 18, 22, 23
*Carcinus maenas*	EGC14	71.36	22.4	16.39	12, 13, 14, 15, 16
*Cancer productus*	RRC1	162.03	42.41	41.8	37, 38, 39, 40, 34, 35, 44, 45, 46
*Cancer productus*	RRC2	150.41	39.49	39.31	41, 42, 47, 48
*Cancer productus*	RRC4	145.57	37.74	33.43	33
*Cancer productus*	RRC5	144.35	36.03	35.58	43, 36

*Note*: The identify of each snail that each crab sublethally damaged is also included.

### 2.2 Prey

Approximately 300 black turban snails (*Tegula funebralis*; Figure 1) were collected from Eagle (Scott's) Bay in Bamfield, BC on September 26, 2022. *T. funebralis* are an ideal model prey species as they are a common prey item for crabs, have roughly uniform shell forms between individuals, and have been well studied in terms of the relationship to repair scars and crabs, such as *C. productus* (Barclay & Leighton, 2022; Molinaro et al., 2014; Stafford, Tyler, & Leighton, 2015; Tyler et al., 2019). The natural range of *T. funebralis* does not overlap with the collection locations of the specific individual crab used in these experiments, ensuring equal “naivete” of each crab to these prey during experiments. Each snail was uniquely labelled using lettered and numbered wire markers affixed to the shell opposite the aperture. Prior to trials, the maximum height (measured from apex to lowest point on aperture) and width (maximum width across the shell perpendicular to the axis of coiling) of each snail was measured using a digital callipers (±0.01 mm). Snails were kept in a tank with continuous water flow and an air stone and continuous access to kelp for food.

### 2.3 Trials

Controlled feeding trials were conducted in which individual crabs of each species were allowed to attack *T. funebralis* and potentially produce predation traces that could become repair scars. The room was kept dark with only a red light source, allowing observation of the crabs without disturbing their behaviour, as crabs are not able to see well under red light conditions (Cronin & Forward, 1988). Prior to the start of each experimental trial, a crab was placed in a 175 L experimental tank for a 20‐min acclimatisation period. Six snails were then placed evenly throughout the tank. For each trial, snails were grouped based on similarity in size to avoid any potential prey preference by the crabs due to snail size. Crabs were allowed to attack individual snails and the duration of all encounters was recorded. Detailed observations of crab predation behaviour were recorded to account for any potential differences in attack strategies that may have influenced the results. Snails were removed from the tank after the initial encounter/attack from a crab. In a few instances, snails were also taken from the crabs when it was clear that no additional shell damage was being created, but the snail was still alive after 20 min. If the crab successfully crushed a snail it encountered, it was left to consume the snail tissue so as not to disturb the crab more than necessary. Shell pieces were removed from the tank once the crab consumed all the tissue and moved away from the attack site. A trial was ended either when the crab caused damage to all the snails or more than 40 min passed without the crab attacking a snail or showing interest in hunting. After each trial, all snails that acquired shell damage from the crab were photographed: three replicate photos each of the snail positioned with the aperture facing left at a 90° angle to the camera lens (apertural view). For each set of photo replicates, the one with the most consistent alignment (aperture perpendicular to the camera) was selected for analysis.

### 2.4 Data analysis

**FIGURE 2:**
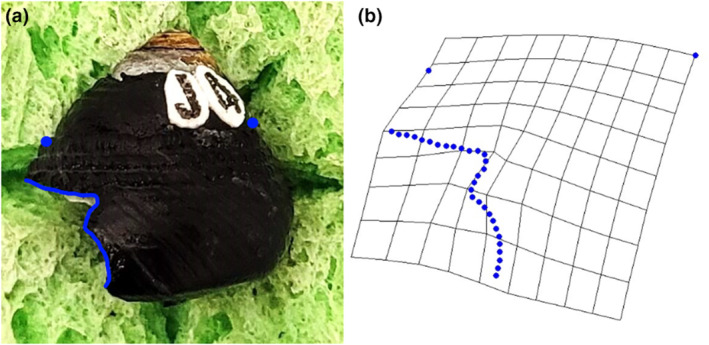
Example of shell damage on *Tegula funebralis* with shell damage outlined and two framing landmarks (top of apertural lip and top of shoulder on opposite side of snail). (a) Subsequent semi‐landmarks of the shell damage created by a crab used for geometric morphometric analyses (b). The grid in (b) is a thin‐plate‐spline showing the distortion of the shape from the overall consensus shape of the full dataset.

The software tpsUtil (Rohlf, 2015) was used to convert all images of snails with shell damage into a TPS file package used in digitising landmarks from photographs. The TPS file was loaded into tpsdig2 (Rohlf, 2015) and the scale of all images was set. For each image, the largest continual shell damage where the damage deviated from the leading edge of the aperture was outlined from either end. Each outline was then converted to 30 evenly spaced semi‐landmarks (Figure 2). Two additional landmarks were chosen to help frame the outlined and semi‐landmarked shell damage, including the top of the apertural lip/leading edge of the aperture (a homologous point), and the top of the shoulder on the opposite side of the snail (Figure 2). The semi‐landmarked photos were saved as a new TPS file that was uploaded into tpsRelw (Rohlf, 2015) to obtain the consensus configuration (least‐squares Procrustes average) of all specimens. After the consensus configuration was calculated, partial and then relative warp analyses were conducted. The relative warp analysis produces axes scores that indicate how each component (warp) contributes to overall changes in shape, (i.e., to evaluate how semi‐landmarks for each specimen differed from the consensus configuration; Rohlf, 2015; Zelditch et al., 2004). Warp axes are assigned loading scores (eigenvalues) that indicate the relative contribution of each axis to the overall observed variation in shape of the dataset. Thin‐plate splines were then used to visually examine the major relative warp axes to determine how scar shapes differed between the three crab species along those warp axes (Figure 2).

Relative warp scores of the major warp axes were then grouped by species and entered into PAST (Hammer et al., 2001) for data analysis. A MANOVA was run on the primary and secondary axes scores to determine if there were any differences between the scar shape produced by each species (where each species fell along an axis of shape change). A linear discriminant analysis (LDA) was conducted to determine how distinct the scar shape of each species was along the major axes of shape change. Discriminant function analyses essentially draw separations/lines between categorised (in this case, to species) samples and then indicate the percentage of individuals in the dataset that were correctly identified to their group (e.g., how often did a *C. productus* specimen plot with the rest of the *C. productus* vs. the *C. maenas* specimens, etc.).

## 3 RESULTS

**FIGURE 3:**
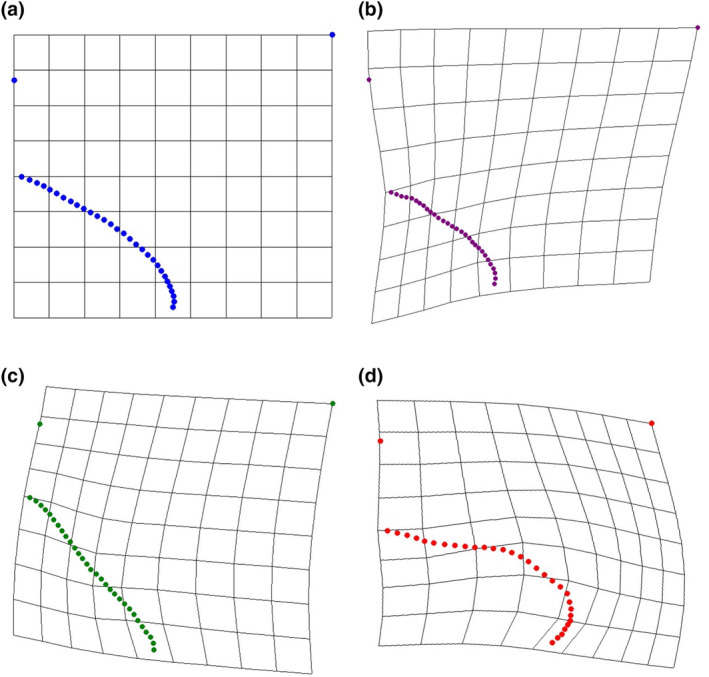
Thin plate splines showing visual trends in shape change in the scars generated by each crab species. (a) is the consensus configuration of entire dataset. (b–d) are consensus shapes of individual species (*Metacarcinus magister*, *Carcinus maenas*, and *Cancer productus*, respectively). These images were generated for visual comparison only and individual species consensus configurations were not used in any statistical analyses.

**FIGURE 4:**
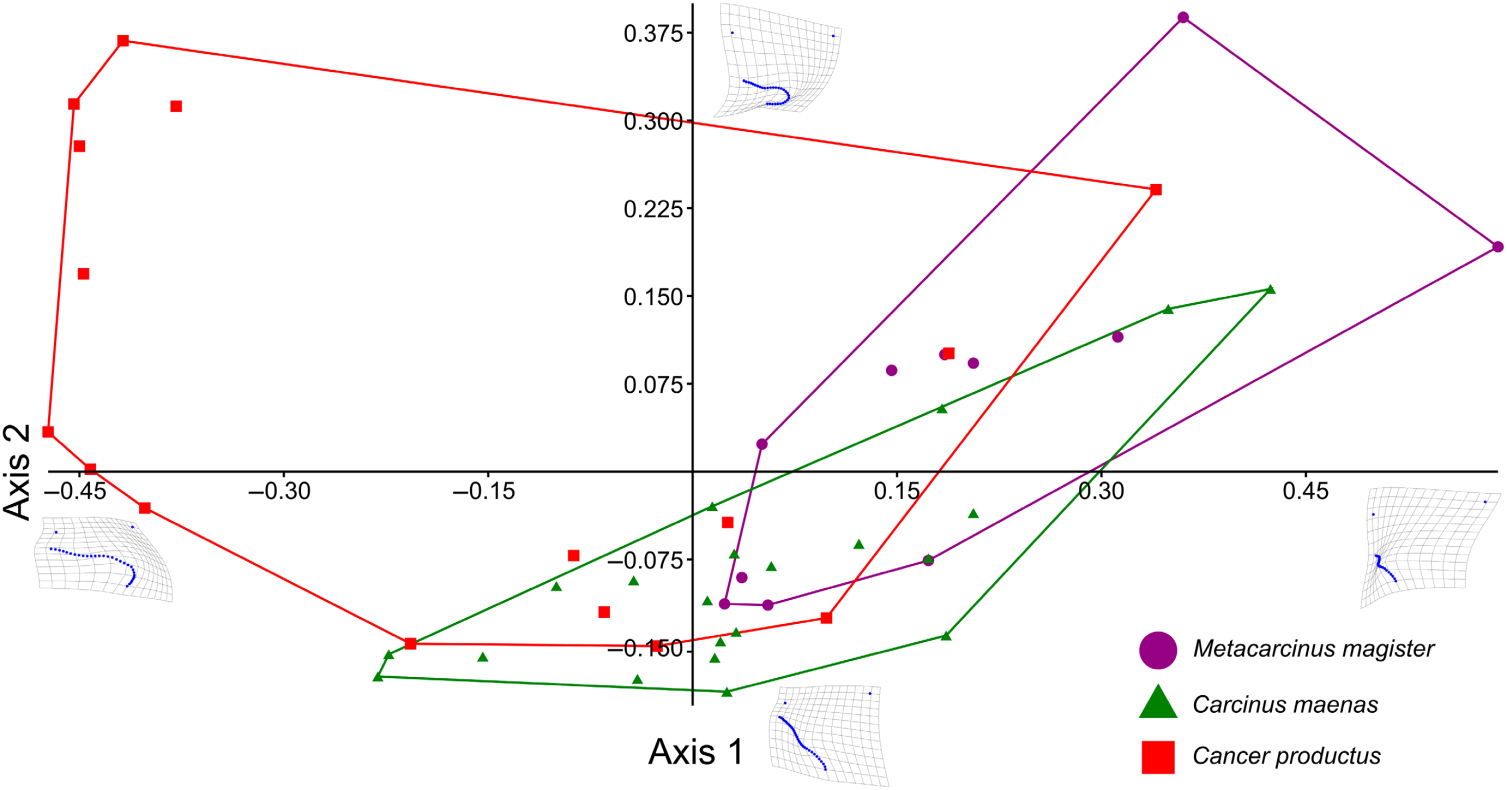
Scatterplot of relative warp scores of Axis 1 and 2. Thin plate spline images on the end of each axis represent the extreme dimensions of shell damage shape change on either end of the axis used to help visually assess how shape is changing (e.g., Axis 1 changes from narrow/deep on the left side of the axis to wide/shallow on the right side of the axis).

**FIGURE 5:**
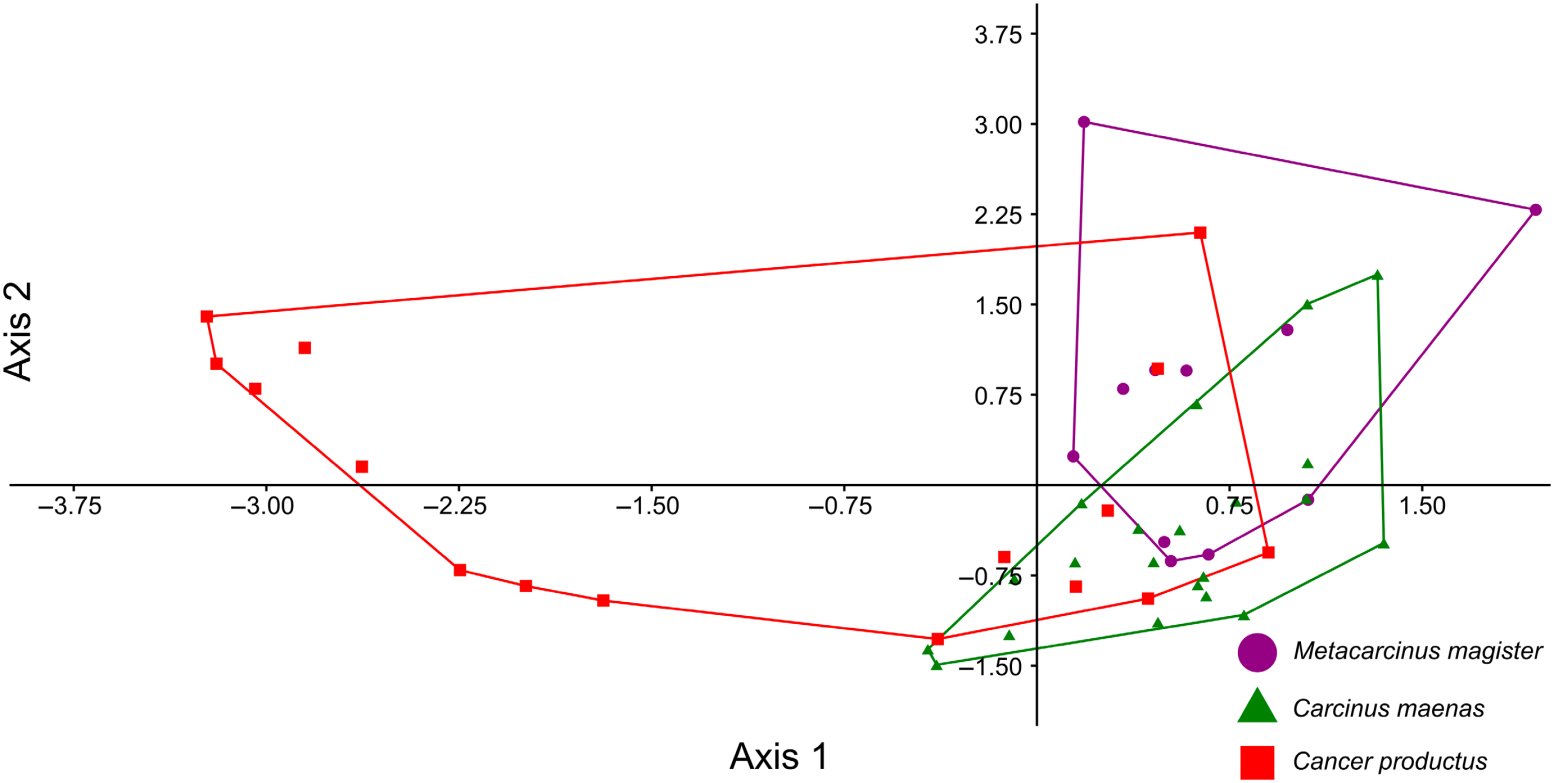
Discriminant function analysis ordination of relative warp scores for Axes 1 and 2 indicating differences in how the shell damage created by each crab species plotted in the ordination space. Points with no overlap between group clusters (convex hull outlines) indicate those that were more likely to be correctly classified within their groups, whereas points in areas of overlap between the species could be incorrectly assigned to other species (see confusion matrix in Table 2).

After all trials were conducted, 48 attacks (*n* = 11, 21, 16 for *M. magister*, *C.maenas*, and *C. productus*, respectively) resulted in sublethal shell damage that could be photographed for geometric morphometric analyses (Tables 1 and A1). The first two warp axes had eigenvalues >1, indicating that these axes contributed more to explaining shape variation than did any individual semilandmark. These two axes accounted for the majority of the overall shape change (82.16%). Subsequent axes contributed 9% or less to the observed shape changes and indicated no visual differences between species; as such, only the first two axes were kept for subsequent analysis. Based on the scores from the first two axes, all three crab species produced uniquely shaped shell damage (MANOVA, *p* < .0001, Table 2). The primary relative warp axis (Axis 1) described a general change in the extent of the damage or how much shell material was removed from the apertural lip, both in terms of overall width and depth/angle of invagination of the damage, and accounted for 52.58% of the total observed shape variation of the shell damage (Figures 3 and 4). The secondary relative warp axis (Axis 2) accounted for 20.53% of the total observed shape variation of the shell damage and roughly depicts a change in the depth/width of the shell damage from wide/shallow to narrow/deep (Figure 4). Shell damage caused by *C. productus* along Axes 1 and 2 was more extensive and deeper than that caused by both other species, with *C. maenas* damage plotting as wide, but shallow, and *M. magister* plotting as an intermediate form (Table 2, Figures 3 and 4). Based on the first two warp axes, 66.67% of the shell damage was correctly assigned to their respective crab species groupings (Table 2, Figure 5).

**TABLE 2:** MANOVA and LDA results, including pairwise comparisons of crab species and a confusion matrix indicating how often each species was correctly classified.

MANOVA *p* (same) < .0001			
*F* = 9.524, df1 = 4, df2 = 88			
Pairwise comparisons (*p*‐values)			
	*Metacarcinus magister*	*Carcinus maenas*	*Cancer productus*
*Metacarcinus magister*		0.0231	0.0003
*Carcinus maenas*	0.0231		0.0001
*Cancer productus*	0.0003	0.0001	

Linear discriminant analysis (LDA)				
66.67% correctly classified				
Confusion matrix				
	*Metacarcinus magister*	*Carcinus maenas*	*Cancer productus*	Total
*Metacarcinus magister*	7	4	0	11
*Carcinus maenas*	4	17	0	21
*Cancer productus*	2	6	8	16
Total	13	27	8	48

### 3.1 Species‐specific predation observations

There were some differences in predation behaviour between species observed during experiments that while not easily testable, may have influenced our results and what is likely to be observed in natural settings. We therefore include these observations for transparency and to inform future work. Crushing was the typical starting strategy for all crab species when attacking a snail. *C. productus* often switched to trying to crush the apex of the shell if they were unable to crush the entire snail before switching to peeling. To peel, *C. productus* grabbed the apertural lip with both claws inserted into the aperture and flexing the apertural lip in opposite directions while stabilising the snail against the ground and with the first two pairs of walking legs. In contrast, *M. magister* often still had one claw grabbing around the entire snail and one claw grasping the apertural lip while peeling. *M. magister* also mostly used their claws to attack the snail and only occasionally also used their first pair of walking legs. *M. magister* crabs also seemed to have a hard time grasping and manipulating the snails and often dropped or struggled to pick up the snails. Instead of peeling, *C. maenas* attempted to crush the apertural lip with their claw around and parallel with the apertural lip as their claws were typically not big enough to try and crush the entire shell. They sometimes used their first set of legs to help stabilise or position the snail. *C. productus* encounters were typically the longest and often either resulted in fatal damage to the shells, or were ended after 20 min of no additional shell damage, despite the unwillingness of the crab to release the snail. In contrast, *M. magister* typically did not take more than 10 min trying to attack any one snail and often abandoned the snails in less time. *M. magister* crabs only made contact with a maximum of three snails before becoming disinterested in hunting or simply sitting still. *C. maenas* were instead quick to attack several snails, often abandoning a snail after a few minutes or less and moving on to attack another snail, sometimes returning to the previous snail. Neither *M. magister* or *C. maenas* killed a snail. *C. productus* and *M. magister* moved mostly along the edges of the tank when not actively attacking a snail whereas *C. maenas* moved more and crossed the middle of the tank much more often. These differences in attacks partially contributed to a difference in the resulting sample sizes between groups, with 16, 11, and 23 damaged shells for subsequent analyses generated by *C. productus*, *M. magister*, and *C. maenas*, respectively (Table A1).

## 4 DISCUSSION

Our results indicate that crabs create scars on their prey that can be attributed to individual crab species, revealing a new method that could be used to reconstruct species‐specific crab abundances both spatially and temporally, monitor the ecological impacts of *C. maenas*, and enhance co‐management strategies of both native crab species and invasive *C. maenas*. We demonstrate that shell damage created by *C. productus* will typically be very deeply invaginated, whereas *C. maenas* shell damage is more likely to be shallow and wide without having any distinct invagination (Figure 3). Shell damage caused by *M. magister* represents an intermediate form that typically is shallowly, but widely invaginated (Figure 3). Paired with a basic understanding of the natural biology, habitat preferences and observed attack strategies, our method provides an alternative, cost‐effective means to track approximate abundances and ecological impacts of three important west coast crab species with a predictable level of certainty.

The differences between the shape of shell damage created by each crab species are likely due to a combination of claw strength and hunting/attack strategies. While there are differences in the size range of these three crab species that will certainly influence their ability to handle prey in natural settings, our results indicate that a crab's ability to peel or chip at the marginal edge of their prey, as dictated by their claw shape and strength (Figure 1), produces characteristic shell damage unique to each crab species. For example, *C. productus* have stronger claws than *M. magister* (Taylor, 2000). When unable to crush a snail, *C. productus* are also known to spend a significant amount of time attempting to peel the shell (Mendonca, 2020), producing deeply invaginated shell damage (Barclay et al., 2020; Boulding et al., 1999), as was observed here. *M. magister* in our experimental trials did demonstrate peeling, but they were quick to abandon those attempts, resulting in less extensive shell damage. Peels were also created differently between these two species. *C. productus* peeled by flexing the apertural lip with the tips of both claws inserted into the aperture, whereas *M. magister* would hold the snail in one claw and attempt to break the apertural lip with the other claw inserted into the aperture. Unlike the other two species, *C. maenas* did not attempt to peel at all, even though *C. maenas* claws are stronger than those of similarly sized *M. magister* (Behrens Yamada et al., 2010). Instead, *C. maenas* shell damage was created by chipping of the aperture as the result of crushing attempts on the apertural lip, rather than the damage caused by flexing of the apertural lip during shell peeling, as was observed with the other two species. The resulting shell damage created by these three species therefore forms a gradient from shallow chipping of the apertural margin from *C. maenas* apertural crushing attempts, to a combination of small peels and shallow chipping of the apertural margin caused by *M. magister* peeling with one claw and holding/crushing the apertural margin, to deep peels from *C. productus* flexing and breaking the apertural margin with both claws. Despite some overlap between the damage created by each species, the amount of variation explained by the first (and second) axis scores is quite strong in terms of geometric morphometric studies, reflecting the natural differences in the strength and attack strategies of each species.

Knowing that shell damage can be attributed to specific crab species has important implications for reconstructing long‐term trends in the relative abundance of these crab species and potentially for *C. maenas* detection and monitoring efforts. Surveys of prey, such as *T. funebralis*, can be conducted easily and quickly from shore during low tides, saving on time and costs associated with trapping and surveying crabs from boats. Prey could be assessed for shell damage and photographed in the field and photographs could be analysed later. More powerfully, simple visual analysis of shell damage may also allow researchers to make immediate informed assessments of which crabs are present in a surveyed area. By applying the methods used here, new datasets could be assessed using the many freely available geometric morphometric software packages, including those used in this study (Rohlf, 2015; https://sbmorphometrics.org/). After using these software packages to calculate relative warp scores of the surveyed specimens, it is then possible to conduct an LDA to determine whether each specimen was assigned to its correct grouping and to what degree of confidence.

In addition to tracking current crab abundances and the ecological impacts of *C. maenas* invasions, repair scars can be used to reconstruct past populations of crabs, complementing accounts from Indigenous knowledge holders (Ban et al., 2017), and providing missing context and baselines for assessing current crab population stocks. As there are few historical records of crabs and given that crabs have a thin exoskeleton and low preservation potential compared to their calcium carbonate shelled mollusc prey, records of repair scars extending back into the fossil record would provide missing long‐term context of how crab populations have changed through time (e.g., Barclay & Leighton, 2022). Historical, archaeological, and fossil records of repair scars could therefore be compared to current prey populations, particularly in areas that have been extensively fished in recent decades, to assess the long‐term impacts of commercial crab fishing.

While *T. funebralis* was chosen for this study because its relationship with crab predation and repair scars has already been well established (Barclay & Leighton, 2022; Molinaro et al., 2014; Stafford, Tyler, & Leighton, 2015; Tyler et al., 2019), this method could easily be extended to assessments of other prey, such as commercially relevant clams and other bivalves, particularly in areas outside of *T. funebralis*'s natural range (e.g., most of the Salish Sea). Repeat surveys could be conducted at regular intervals to also track the abundance of different crab scars, as the frequency of scarred prey individuals provides a proxy for relative crab abundance (i.e., more scars indicates more crabs and vice versa; Stafford, Tyler, & Leighton, 2015). However, it is important to consider the lifespan of the prey, as damage from crabs is accumulated over the lifetime of the prey, forming signals that can be decadal (Tyler et al., 2019). Tagging of surveyed individuals and only assessing recent growth on prey for repair scars would therefore be an ideal approach to repeat surveys. As with any biological system, there is some overlap between the marks generated by the three crab species, but by implementing a geometric morphometric approach, it is possible to quantify the level of certainty in each identification. Repair scar surveys on multiple prey species would also provide a new tool to investigate the impacts of crab predation, particularly by invasive *C. maenas*, on prey populations through time.

*Carcinus maenas* are very successful and damaging invaders that represent a major priority for many local communities and NPOs, as well as federal, provincial, and state agencies. However, current ongoing detection and monitoring of *C. maenas* invasions is very expensive and time‐consuming. Our method adds to a growing body of co‐management strategies, such as incorporation of Indigenous and local community knowledge, that could be included to help minimise the impacts of *C. maenas* invasions and associated mitigations costs. Given that the strongest differences in shell damage shape were observed between *C. maenas* and *C. productus*, our method would be particularly effective for intertidal, rocky shores, or any other areas where there are likely to be few *M. magister*. Repeat surveys could also be used to track the relative abundance of different crab species and assess the impacts of *C. maenas* invasions on both native mollusc prey and crab species. For example, as *C. productus* are known to prey upon *C. maenas* (Hunt & Behrens Yamada, 2003; Jensen et al., 2007), repeat surveys could be used to determine how effective *C. productus* are as a deterrent to *C. maenas* and to assess the impacts of *C. maenas* predation and densities on mollusc prey. Repair scar surveys could also be paired with other assessments of species threatened by *C. maenas* invasion, such as eelgrass habitats (Howard et al., 2019), to assess and monitor the impacts of *C. maenas* presence and/or abundance on the broader ecosystems which they invade.

Using the shape of repair scars to distinguish between crab species also has useful implications for *M. magister* and *C. productus* fisheries. Surveys of repair scars could be conducted to provide rapid assessments of crab populations with broader spatial coverage that could supplement more expensive and time‐consuming crab trapping surveys, and could be compared to trends observed from historical, archaeological, or fossil studies of repair scars. Even though *M. magister* is the primary target for commercial crab fishing, crabs are typically managed as a multispecies fishery. The ability to distinguish between crab species could also therefore provide a useful means to assess the impacts of commercial and recreational crab fishing on *M. magister* and *C. productus* separately.

While *M. magister*, *C. maenas*, and *C. productus* are not the only species of shell crushing crabs found on the west coast of North America, our study represents an important innovation for assessing these three important and abundant crab species. Further studies could develop additional geometric morphometric assessments of crab shell damage created by crabs such as the graceful rock crab (*Metacarcinus gracilis*), or shell damage on other common prey species, such as clams and other gastropods. We conclude that geometric morphometric assessments of repair scars on crab prey provide a new, cost‐effective and easily implemented method for understanding the ecological impacts of *C. maenas* invasions, and for tracking crab populations at the species level, across broad spatial and temporal scales.

## AUTHOR CONTRIBUTIONS

**Kristina M. Barclay:** Conceptualization (lead); data curation (supporting); formal analysis (lead); funding acquisition (lead); investigation (supporting); methodology (lead); supervision (lead); writing – original draft (lead); writing – review and editing (lead). **Paige Amos:** Data curation (equal); formal analysis (supporting); funding acquisition (equal); investigation (lead); methodology (supporting); writing – original draft (supporting); writing – review and editing (equal). **Lindsey R. Leighton:** Conceptualization (equal); formal analysis (supporting); methodology (supporting); writing – review and editing (equal). **Chris L. Schneider:** Conceptualization (equal); writing – review and editing (equal). **Julia K. Baum:** Funding acquisition (supporting); supervision (supporting); writing – review and editing (equal).

## CONFLICT OF INTEREST STATEMENT

The authors have no competing interests.

## Supporting information


Data S1.

## Data Availability

All data are available in the manuscript and Data S1.

## APPENDIX A

**TABLE A1:** Summary of snails that had sublethal damage during experimental trials and their relative warp scores generated from geometric morphometric analyses. Relative warp scores were grouped by crab species for subsequent statistical analyses.

Snail	Snail height (mm)	Snail width (mm)	Crab ID	Crab species	Axis 1	Axis 2
1	23.64	21.89	D3	*Metacarcinus magister*	3.61E‐02	−9.05E‐02
2	19.89	19.57	D3	*Metacarcinus magister*	2.34E‐02	−1.13E‐01
3	17.11	19.01	D1	*Metacarcinus magister*	5.09E‐02	2.35E‐02
4	17.5	17.91	D1	*Metacarcinus magister*	1.46E‐01	8.65E‐02
5	17.57	16.86	D1	*Metacarcinus magister*	5.91E‐01	1.92E‐01
6	17.24	18.47	D1	*Metacarcinus magister*	2.06E‐01	9.26E‐02
7	20.86	20.38	D3	*Metacarcinus magister*	3.12E‐01	1.15E‐01
8	20.94	20.42	D3	*Metacarcinus magister*	1.73E‐01	−7.59E‐02
9	20.14	19.77	D3	*Metacarcinus magister*	5.52E‐02	−1.14E‐01
10	20.58	21.15	D2	*Metacarcinus magister*	1.85E‐01	1.00E‐01
11	17.21	18.52	D2	*Metacarcinus magister*	3.60E‐01	3.88E‐01
12	16.97	17.54	EGC14	*Carcinus maenas*	1.83E‐01	5.40E‐02
13	16.16	16.2	EGC14	*Carcinus maenas*	−2.23E‐01	−1.56E‐01
14	16.97	17.4	EGC14	*Carcinus maenas*	1.08E‐02	−1.10E‐01
15	15.18	16.61	EGC14	*Carcinus maenas*	−1.00E‐01	−9.77E‐02
16	14.3	15.44	EGC14	*Carcinus maenas*	−2.31E‐01	−1.75E‐01
17	19.46	19.55	EGC10	*Carcinus maenas*	4.24E‐01	1.56E‐01
18	20.96	20.29	EGC10	*Carcinus maenas*	2.06E‐01	−3.54E‐02
19	18.95	18.78	EGC5	*Carcinus maenas*	1.73E‐01	−7.40E‐02
20	18.69	19.44	EGC5	*Carcinus maenas*	2.51E‐02	−1.88E‐01
21	18.15	18.45	EGC5	*Carcinus maenas*	1.22E‐01	−6.17E‐02
22	20.34	19.86	EGC10	*Carcinus maenas*	3.19E‐02	−1.37E‐01
23	20.65	19.64	EGC10	*Carcinus maenas*	5.77E‐02	−8.06E‐02
24	18.03	18.4	EGC6	*Carcinus maenas*	1.86E‐01	−1.40E‐01
25	16.03	16.4	EGC6	*Carcinus maenas*	3.05E‐02	−7.00E‐02
26	17.77	17.41	EGC6	*Carcinus maenas*	1.44E‐02	−2.96E‐02
27	17.12	17.77	EGC6	*Carcinus maenas*	3.49E‐01	1.39E‐01
28	18.37	19.11	EGC1	*Carcinus maenas*	−1.54E‐01	−1.58E‐01
29	19.52	19.11	EGC1	*Carcinus maenas*	1.62E‐02	−1.59E‐01
30	19.78	18.87	EGC1	*Carcinus maenas*	−4.33E‐02	−9.29E‐02
31	19.22	18.89	EGC1	*Carcinus maenas*	−4.05E‐02	−1.77E‐01
32	17.98	18.73	EGC1	*Carcinus maenas*	2.04E‐02	−1.45E‐01
33	22.09	21.64	RRC4	*Cancer productus*	−2.07E‐01	−1.47E‐01
34	19.28	19.95	RRC1	*Cancer productus*	1.88E‐01	1.01E‐01
35	20.25	21.28	RRC1	*Cancer productus*	−4.73E‐01	3.39E‐02
36	17.32	17.84	RRC5	*Cancer productus*	−2.61E‐02	−1.49E‐01
37	21.46	19.95	RRC1	*Cancer productus*	3.40E‐01	2.41E‐01
38	19.54	19.35	RRC1	*Cancer productus*	−4.02E‐01	−3.11E‐02
39	19.84	18.88	RRC1	*Cancer productus*	−3.79E‐01	3.12E‐01
40	20.22	20.54	RRC1	*Cancer productus*	−4.50E‐01	2.78E‐01
41	18.96	19.38	RRC2	*Cancer productus*	−8.73E‐02	−7.17E‐02
42	18.09	17.33	RRC2	*Cancer productus*	2.57E‐02	−4.35E‐02
43	16.82	16.76	RRC5	*Cancer productus*	9.82E‐02	−1.25E‐01
44	22.06	23.43	RRC1	*Cancer productus*	−4.42E‐01	2.01E‐03
45	20.19	21.56	RRC1	*Cancer productus*	−4.47E‐01	1.69E‐01
46	23.2	23.7	RRC1	*Cancer productus*	−4.18E‐01	3.68E‐01
47	20.87	21.86	RRC2	*Cancer productus*	−6.49E‐02	−1.20E‐01
48	20.42	20.67	RRC2	*Cancer productus*	−4.54E‐01	3.14E‐01
